# Adoption of an innovation to repair aortic aneurysms at a Canadian hospital: a qualitative case study and evaluation

**DOI:** 10.1186/1472-6963-7-182

**Published:** 2007-11-15

**Authors:** Nathalie M Danjoux, Douglas K Martin, Pascale N Lehoux, Julie L Harnish, Randi Zlotnik Shaul, Mark Bernstein, David R Urbach

**Affiliations:** 1 Department of Health, Policy, Management and Evaluation, University of Toronto, Toronto, Canada; 2 Department of Health Administration, University of Montreal, Montreal, Canada; 3 Division of Clinical Decision-making and Health Care, University Health Network, Toronto, Canada; 4 Department of Pediatrics, University of Toronto, Toronto, Canada; 5 Department of Surgery, University of Toronto, Toronto, Canada; 6 Joint Centre for Bioethics, University of Toronto, Toronto, Canada; 7 Bioethics Department, The Hospital for Sick Children, Toronto, Canada

## Abstract

**Background:**

Priority setting in health care is a challenge because demand for services exceeds available resources. The increasing demand for less invasive surgical procedures by patients, health care institutions and industry, places added pressure on surgeons to acquire the appropriate skills to adopt innovative procedures. Such innovations are often initiated and introduced by surgeons in the hospital setting. Decision-making processes for the adoption of surgical innovations in hospitals have not been well studied and a standard process for their introduction does not exist. The purpose of this study is to describe and evaluate the decision-making process for the adoption of a new technology for repair of abdominal aortic aneurysms (endovascular aneurysm repair [EVAR]) in an academic health sciences centre to better understand how decisions are made for the introduction of surgical innovations at the hospital level.

**Methods:**

A qualitative case study of the decision to adopt EVAR was conducted using a modified thematic analysis of documents and semi-structured interviews. Accountability for Reasonableness was used as a conceptual framework for fairness in priority setting processes in health care organizations.

**Results:**

There were two key decisions regarding EVAR: the decision to adopt the new technology in the hospital and the decision to stop hospital funding. The decision to adopt EVAR was based on perceived improved patient outcomes, safety, and the surgeons' desire to innovate. This decision involved very few stakeholders. The decision to stop funding of EVAR involved all key players and was based on criteria apparent to all those involved, including cost, evidence and hospital priorities. Limited internal communications were made prior to adopting the technology. There was no formal means to appeal the decisions made.

**Conclusion:**

The analysis yielded recommendations for improving future decisions about the adoption of surgical innovations. ese empirical findings will be used with other case studies to help develop guidelines to help decision-makers adopt surgical innovations in Canadian hospitals.

## Background

Innovations in surgery, such as open-heart surgery, organ transplantation and minimally invasive procedures, continue to benefit patients worldwide. A surgical innovation can be defined as a new "*intervention that is not viewed by the institution, community or profession as meeting the accepted standards of safety, reliability and familiarity with effects, side effects and complications*" [1]. In an attempt to improve an existing technique, implement a new technology or enhance institutional productivity, surgical innovations are often introduced by individual surgeons under independent circumstances. As a result of this ad-hoc approach, no mechanism exists to capture and share what has been learned from these experiences [2]. Decision-making processes for the adoption of surgical innovations have not been well studied, and a standard process for the introduction of surgical innovations in hospitals does not exist.

The demand for less invasive approaches to surgical procedures by patients, health care institutions, and industry, places increased pressure on surgeons to acquire the appropriate skills to adopt innovative procedures. Pressure to adopt new technologies within departments can make decision-making for these innovations challenging. Individual surgeons are usually not in a position to make judgments about collective resource allocation, and may exert pressure for the implementation of innovations outside the usual institutional decision-making process. Surgeons often adopt new technologies into practice despite poor evidence regarding the efficacy of an innovation [3]. Even when evidence supports an innovation, practitioners may not always choose to adopt it. For example, percutaneous transluminal coronary angioplasty (PTCA), an alternative to coronary artery bypass grafting that relieves narrowing and obstruction of the coronary arteries, experienced slow adoption rates in some hospitals despite strong supporting evidence [4].

With the emergence and rapid diffusion of new surgical technologies, there is an increasing need to prioritize and contain costs to ensure the sustainability of the health care system [2,5,6]. In addition to requiring that treatments have some scientifically sound basis, [7], public accountability is an important consideration when resources might be diverted from other causes [8]. Decision-makers in Canadian healthcare institutions must balance the benefits of using a new technology against cost and risk to patient safety, in a context where reliable data are scarce. In other words, a method of priority setting is required for the adoption of new surgical technologies [9].

Priority setting is a challenge for every health care system because demand for health services outweighs available resources. As a result, hospital decision-makers are forced to set priorities for providing access to health care services. In every health system, decision-makers must ensure that they achieve two key goals in priority setting: legitimacy, defined as the moral authority to make allocation decisions about available resources [10]; and fairness, which is achieved when an individual has sufficient reason to accept a priority setting decision because of the acceptability of the decision-making process [11]. Legitimacy and fairness are related in that decision-makers' legitimacy may be enhanced by their commitment to the use of a fair decision-making process. Before evaluating the legitimacy and fairness of priority setting, it is beneficial to first understand how these decisions are currently made [12]. Understanding these experiences will provide insight on the varying adoption patterns and provide guidance for addressing the challenge of priority setting in surgery. Surgical innovations face different challenges than other healthcare innovations such as pharmaceutical agents. For example, there is less government regulation of surgical procedures and devices than drugs, and there may be greater budgetary constraints and lack of research to support surgical procedures and devices. Until now, decision-making processes for adopting surgical innovations have not been well studied.

The purpose of this paper is to describe and evaluate the adoption of a new health technology used by surgeons for the treatment of aortic aneurysms called endovascular aneurysm repair (EVAR). This surgical innovation posed unique economic and ethical challenges for the institution. Results from this study will contribute to the development of guidelines to help decision-makers in relation to future surgical innovations in Canadian hospitals.

## Methods

### Study Design

We used qualitative case study research methods to study the introduction of EVAR in an academic health sciences centre. A case study is "*an empirical inquiry that investigates a contemporary phenomenon within its real-life context*" [13]. Since the adoption of surgical procedures is complex, context-dependent and influenced by social processes, these methods are appropriate [14].

### Setting

The case study was conducted at a large, urban university academic health sciences centre with approximately 500 patient beds in Toronto, Ontario, Canada. In the province of Ontario, hospitals are funded by the provincial Ministry of Health and Long-Term Care with an annual global budget. Certain health programs are funded under special agreements between the hospital and the province. Residents of Canada are entitled to universal coverage of all medically necessary health services [15], however, decision-makers in the health system are constantly struggling to determine which services are 'medically necessary' and therefore eligible for publicly funded coverage.

### Case Description

Endovascular Aneurysm Repair (EVAR) is a new surgical technology for repair of abdominal aortic aneurysms (AAA) involving the insertion of a prosthetic graft into the site of an aneurysm using catheters inserted through the femoral artery. An aneurysm is an abnormal dilatation of a blood vessel. AAAs are life threatening because of their potential for rupture. The traditional treatment for high-risk AAAs is surgical repair, with reconstruction of the dilated aorta using prosthetic material. Since these major operations are usually performed in elderly patients with atherosclerosis and other medical co-morbidities, there is a substantial risk of death and serious complications associated with surgery [16]. Endovascular placement of a covered stent through a percutaneous transfemoral approach to exclude the abdominal aortic aneurysm has recently emerged as an alternative to open surgical repair, since it can be done in a radiology suite without a general anesthetic [17,18]. Although the risks, costs and outcomes of this novel approach were uncertain at the time when decisions about adoption were being made in the late nineties [19], some 25,000 had been performed in the United States [18]. Key potential benefits of EVAR were faster recovery and decreased length of hospital stay. Key concerns were its relatively high cost of approximately $10,000 US per device [20-22] and lack of evidence regarding safety and effectiveness.

### Sampling Techniques

Sampling of participants was conducted using purposive and theoretical sampling techniques. Purposive sampling allows us to sample a homogeneous sample of participants who will be able to describe their experiences related to the study question. The first participant selected was the surgeon who introduced the innovation to the hospital. Additional participants were contacted directly through their known involvement with the innovation and others were identified by other interviewees – this tends to result in a homogeneous sample, important in the initial phases of developing the theory [23]. Theoretical sampling involves selecting individual participants based on concepts that emerge from the data as the data are being collected. Analysis was done concurrently with the interviews, which helped in generating new concepts that further directed data collection in subsequent interviews [24]. This combination of sampling techniques helped ensure a diversity of experiences was captured based on actual experience with the innovation. Sampling decisions were made concurrently with data analysis until theoretical saturation was reached, when additional information was not forthcoming from further interviews. The sampling frame included surgeons, nurses, and decision-makers involved in hospital priority setting decisions.

### Data Collection

Interviews were conducted with individuals identified to have the most involvement with the innovation (3 vascular surgeons, 1 hospital decision-maker, 1 radiologist). Two interviewers collected data using a semi-structured interview format with open-ended questions. An interview guide was developed and revised in light of data collected and emerging findings [25]. Four of the interviews were conducted in person and one by teleconference and all were audiotaped. Interview participants were asked to describe their experience with the decision-making process regarding implementation of this surgical innovation. We also analyzed relevant documents including the hospital's Strategic Fiscal Reports.

### Data Analysis

Tape recordings were transcribed verbatim as text documents and then uploaded into software designed for qualitative data management and analysis (QSR International, NVivo 2.0). Analysis consisted of a modified thematic analysis using open, axial and selective coding [25]. Open coding involves familiarization of the researcher with the data and identifying 'chunks' of data that relate to an idea. Data are read line-by-line and coded using a term that best represents the idea. Axial coding involves organizing the codes into thematic categories. Similar ideas were organized into overarching themes. The two interviewers independently coded the first set of interviews and discrepancies were compared and resolved to establish a uniform coding system. The coded transcripts were then sorted into emergent themes and compiled into a single document. These data were used to generate a description of the decision-making process involved in implementing EVAR at the hospital and this description was evaluated by comparing it to the conditions of the '*Accountability for Reasonableness*' framework (described below). Compliance with the framework was considered 'good practice' and instances where the conditions were not met were considered 'opportunities for improvement'.

We used several strategies to enhance the validity of our analysis. Codes connected to original quotations in the source material provided traceability or grounding. As a 'member check', we provided participants with an opportunity to view verbatim transcriptions, written interpretations and reports to confirm their accuracy. To increase the theoretical sensitivity of the study [24], the interviewers reviewed the literature on surgical innovations and the theoretical framework. The interviewers recorded memos, notes and reflective thoughts during periods of data collection and analysis. The data were then transcribed either as short notes or verbatim for periodic discussion with the research team (peer debriefing). Transcripts and documents were dually coded and a mutual coding list was developed to ensure consistency of the analysis. The use of two interviewers allowed the capture of different perspectives. One interviewer took notes during the interview and posed questions throughout as necessary if something was missed or required further exploration. This enhanced the interview data by allowing non-verbal nuances to be described and noted throughout the interview. After the interview, the two interviewers debriefed and discussed ways to improve for the next interview. This technique was particularly useful in the first interview where much exploration occurs.

### Conceptual Framework

The findings from this case were analyzed using the conceptual framework *Accountability for Reasonableness *[11]. *Accountability for Reasonableness *was developed in the context of managed care in the United States, and is a leading framework for analyzing priority setting. It is an ethical framework that operationalizes the abstract concept of fairness, is empirically based, and grounded in theories of democratic deliberation. According to Daniels & Sabin (2002), an institution's priority setting decisions may be considered fair if they satisfy four conditions: *Relevance, Publicity, Appeals and Enforcement *(Table 1).

**Table 1:** Accountability for reasonableness

**Condition**	**Description**
Relevance	Allocation decisions should be based on reasons (e.g. evidence, principles) that fair-minded people can agree are relevant under the circumstances.
Publicity	Allocation decisions and their rationales should be publicly accessible.
Appeals	There should be opportunities to revisit and revise decisions on the basis of new information and there should be a mechanism to resolve dispute.
Enforcement	There should be mechanisms in place to ensure that fair processes are implemented.

The Relevance condition ensures that the rationales for the decisions being made are based on evidence – reasons and principles that fair-minded parties agree are relevant to deciding how to meet given needs under resource constraints. The Publicity condition stipulates that the decisions being made and their rationales must be publicly accessible. The Appeals condition ensures that a mechanism exists for challenging decisions made and dispute resolution regarding limit-setting decisions exists, including the opportunity for revision of decisions when new evidence becomes available. The Enforcement condition ensures public regulation of the process to ensure that the first three conditions are met.

We used the *Accountability for Reasonableness *framework in our analysis to understand the decision-making process for adopting EVAR in an academic health sciences centre, since it identifies the conditions for fair process in limit setting exercises.

### Research Ethics

This study was approved by the Research Ethics Boards at the University Health Network, The Hospital for Sick Children, and the Committee on the Use of Human Subjects at the University of Toronto. Participation in this study was voluntary and written informed consent from each respondent was obtained. All transcripts were kept anonymous and confidential, available only to the research team. No study participants have been identified.

## Results

In this section, we describe and evaluate the implementation of EVAR in the hospital. The first part describes two key decisions regarding EVAR at the hospital: (1) the decision to adopt EVAR in the division of vascular surgery and (2) the decision to stop hospital funding for the technology. The second part evaluates these findings according to the four conditions of *Accountability for Reasonableness*. Verbatim quotes are used to illustrate key points.

### Description of decision-making process

#### Decision to adopt EVAR

There were two stages of implementation of EVAR at the hospital. In 1998, sporadic cases were performed according to individual patient needs. A Surgeon decided to treat these initial patients with EVAR because the patient "*would not tolerate a major operation, yet was in severe pain*". Eleven patients were treated with 'homemade' grafts made by hand for the first patient. The Surgeon explains that "*at the time, there was no graft that was ideal for [the patient]...so my wife and I figured out how to sew plastic on the flaps of the stent with a suture*." This introduction demonstrated the feasibility of the procedure. The Surgeon had knowledge about EVAR because he was involved with the development of a protocol through a specialty medical organization of which he was a prominent member.

By 2001, two Food and Drug Administration (FDA) approved devices were available on the market in the United States, with approvals for selected stents in Canada through Health Canada's Therapeutic Products Directorate (TPD). The TPD is the national authority that regulates, evaluates and monitors the safety, efficacy and quality of therapeutic and diagnostic products available to Canadians [26].

At that time, EVAR was approved under the hospital's global budget, and an additional 85 procedures were performed. A vascular surgeon, who had been trained in the technique, was recruited specifically to develop the endovascular program at the hospital. He explained that he agreed to join the hospital because there were appropriate funds committed to an EVAR program at the time. Another surgeon in the department described his initial experience with the procedure.

"*We practiced just watching what we were doing, then we did it under x-ray control...then we had a couple of experts come and we did it with them on two patients the next day*."

EVAR was offered to patients who were thought to be poor surgical risks. One surgeon commented that a patient had a high risk of dying from conventional surgery and therefore wanted to perform a procedure that had a low risk. There was a perception on the part of surgeons that frail elderly patients and those with co-morbidities would benefit from quicker recovery periods associated with less invasive surgery.

"*The difference in mortality from an open operation versus [EVAR] is SO huge...the difference between doing open operations and endograft are astronomical. Mortality rates are 3% versus 17 or 18%*."

The surgeons were confident about the benefits of this innovation, even though data on safety and effectiveness were preliminary and only short-term follow-up information was available.

When surgeons were questioned regarding introducing new technologies, they all described their desire to introduce new techniques and innovative approaches in their practices given the opportunity, particularly given that they were in an academic centre, which encourages innovation. This was true for the introduction of EVAR which was an innovative approach that seemed to 'make sense' for the patient and was therefore adopted within the department.

The Radiology department was involved because initially the procedures, which required fluoroscopic control, were done in the angiography suite. However, after the first few had been performed, the procedure was moved to the Operating Room (OR). This move had a greater impact on radiology since they needed more staff to run their regular activities as well as attend the OR. There was discussion between the surgical team and radiology about (1) the best place to perform the procedure; and (2) who, between the surgeons and radiologists, should perform it. Radiology had better imaging capability than the OR, however, in the event of complications, it was felt that a patient could be treated more safely in the OR. EVAR was a politically challenging undertaking, which exposed conflicting views between clinicians about endovascular leadership.

Radiologists felt the patient was better treated in the angiography suite and surgeons felt the OR was most suitable. One radiologist expressed that:

"*Nursing services didn't like to have to bring bodies down here outside the OR, anesthesia didn't like it, the surgeons didn't like it...people didn't like moving from where they were...and since there were three of them and one of us...that speaks for itself*."

Differing views about the roles and responsibilities of different physicians were revealed in the interviews. The vascular surgeons felt they were 'held hostage' by radiology. The conflicting views held by both sides demonstrated disagreement about 'ownership' of the innovation, and the willingness of 'competing' providers to communicate and collaborate. A surgeon commented that:

"*A lot of the other practitioners of catheter-based techniques, such as the radiologists, were very unhappy that someone else could come and do this...they were present in the operating room but in essence because of the way the hospital structured it and was unwilling to commit completely, so we were held hostage by them...In fact, the tech that worked our machine worked for them and so they could essentially withhold him, and would if they were not allowed to actively participate. But in terms of expertise with regard to endovascular aneurysms, unfortunately, they did not bring much*."

Due to the desire to retain 'ownership' and control of the innovation, the vascular surgeons were not willing to 'give up' their patients to the radiologists, who they felt did not have expertise with management of aortic aneurysms.

#### Decision to stop funding

In 2004, the hospital stopped funding EVAR, seeking specific financial support from the Ontario Ministry of Health and Long-Term Care. The device cost of approximately $10,000 US was the determining factor, even though costs of open AAA repair are probably very similar to the overall cost of EVAR [27]. The hospital, which was funded by a global budget, was less willing to fund incremental use of a new technology than hospitals funded under different models, such as fee for service models of Diagnosis Related Groups (DRG) as occurs in hospitals in other countries such as the United States. The vascular surgeons were upset with this decision, feeling that the hospital was using the department as leverage to force the province to pay for the procedure. One surgeon explained that:

"* [The hospital decided to] pull all the endovascular funding and used the department, our division specifically, as pawns in a little game or a little tug of war they were playing with the provincial government in trying to gain additional funding*."

It was explained that the decision was strictly 'financial' due to the high costs associated with the stents. The hospital had decided that their budget would no longer include new procedures that were not fully funded by the government. The hospital decision-maker explained:

"*This clearly is a very expensive technology. It can be fifteen thousand dollars just to put in stents for one patient [and the government] should be funding it...As we were getting into progressive budget cuts, we decided that realistically it didn't make sense for us to continue doing it, given that we decided on a budget policy in the organization that we shouldn't be doing new things unless they were fully funded by the government. So this was something we'd already started doing which wasn't funded by the government*."

Although EVAR was already being funded by the hospital, it was explained that the hospital had just added it on one year, on an ad hoc basis, and its continued use had to be re-evaluated. The decision-maker said that it was initially introduced because a hospital decision-maker at the time was a vascular surgeon who advocated on behalf of its implementation. Ultimately, the re-evaluation occurred due to a prioritization process designed to set the institution's future goals. The hospital decision-maker explained:

"*I think it sneaked into the organization quite honestly, just around the time that I arrived here...the person who was the [hospital decision-maker] at the time was a vascular surgeon by background. Seeing that it was essential to the future of vascular surgery, [he] found a way of absorbing it in his budget but it started to become a financial problem for us. Plus, when we did our clinical activity target setting as part of our strategic planning, we actually decided that vascular surgery was not a real priority for us*."

The vascular surgeons were upset by the decision to stop EVAR. They expressed concern about patients who were now faced with an increased burden of traveling to another city for the procedure.

"*All the while that this was happening we had a couple of patients die waiting for aneurysm repairs. We had to transfer patients to [other city] and whereas it might be fine for you or I to go to [other city] to get treatment, when you're 80 years old, that's not a terribly reasonable thing to have happen to you because of the logistics of the difficulties involved. So there were many adverse events that resulted because of this*".

### Evaluation of decision-making using 'Accountability for Reasonableness'

#### Relevance Condition

The decision to adopt EVAR was based on two reasons. First, all respondents stated that their perception of improved patient outcomes and safety was the primary criterion for adoption. AAAs are common and their treatment can sometimes result in a hospital stay of approximately ten days. Less invasive options were preferable, particularly for patients who had multiple medical problems, or were elderly and poor surgical risks. One surgeon explained:

"*If you have an incision [and] if you have an aneurysm in the chest, it may not be obvious to you or to a surgeon who doesn't work in the chest, because to work in the chest you have to open the chest which means the lungs collapse, which means you're working on one lung or less...the whole middle of the chest can collapse and move and indeed does, and collapse your other lung so if you open this chest you only have one lung right off*."

Second, due to a drive to innovate, surgeons were eager to adopt innovations that their colleagues at other institutions were using.

"*You shouldn't have to scream every time you need something and you shouldn't have to have people die in order to get what would be the standard of care in the rest of the world to which we aspire to compare ourselves*."

A surgeon was hired specifically to perform endovascular repairs and was instrumental to its early use.

"*The thing that really kick started it is when we hired a partner who had been trained to do this in the US. So he had done a lot of these cases, and so we then went fairly rapidly and ramped up the program*."

The decision to stop EVAR was based on three reasons. The first reason was cost. One surgeon explained that the department "*ran out of money early because we had an inadequate amount of money to fund the volume of surgery which we were expected to do*." Subsequently, the hospital Business Planning Brief Fiscal 2004/05 stated that:

"*Pending the results from the Ministry's review of vascular studies, [hospital] will not be doing any endovascular cases during this fiscal year unless specific volume based funding for stents becomes available*".

Another surgeon explained the impact this had on their staff. The surgeon hired to perform EVAR left the hospital shortly after the budget was cut.

"*If you don't give [surgeons] the tools to do their job, they're going to leave. And that's exactly what happened. [The Surgeon] left. They cut the budget, he said 'fine I'm outta here'. It took him six months and he was gone*."

Second, there was concern that evidence to support the safety and effectiveness of EVAR was lacking. The decision-maker explained that this was one of the factors that led to the decision to cut funding, especially since the ministry didn't view it as a proven therapy. A committee mandated to advise Ontario's Ministry of Health and Long-Term Care on the adoption of new health technologies had recommended against widespread use of EVAR until long-term follow-up data from clinical trials were available and recommended that the use of EVAR be limited to a field evaluation at one site in the province [26]. The hospital decision-maker reasoned:

"*We took the position that in a sense it really wasn't even a proven technology in the view of the government otherwise why in the world would they evaluate it? It didn't make much sense for us to be subsidizing out of our base budget something that the government viewed as not even being a proven therapy*".

Third, through their strategic planning process, the institution decided that vascular surgery was not a priority.

From this analysis, the decision to adopt EVAR did not meet the Relevance condition because the reasons supporting its adoption did not involve all the appropriate stakeholders. The decision of the hospital to stop funding EVAR did satisfy the Relevance condition.

#### Publicity Condition

A key hospital decision-maker at the time was approached by one of the vascular surgeons about the procedure and agreed it was important. Communication was also made with the hospital's Research Ethics Board (REB). One Surgeon explained that he spoke "*to the chief of the [REB] committee...and I told him everything and what I was going to do. And he said, 'ok, go ahead'. I just wanted someone to know about it*." This was not systematically publicized throughout the hospital or the community or to the Ministry of Health and Long-Term Care. Communication was made with other departments that were involved with the procedure (such as radiology) however, this communication was made after the vascular surgeons decided to introduce the innovation to the hospital. Patients were informed at the time of their procedure and were aware that EVAR was not considered to be 'standard of care'. One Surgeon explained that "*it sort of came by word of mouth because we weren't out advertising this, out looking for people*."

The decision to stop EVAR became known to the surgeons through communications from a hospital decision-maker to the head of the Division of Vascular Surgery. The public was made aware of this decision through hospital communications reporting the priorities of the hospital and word of mouth that the vascular program was no longer a priority for the hospital.

The decision to adopt EVAR did not satisfy the criteria for the Publicity condition since it did not ensure all stakeholders had an opportunity to engage in the deliberation. The decision to stop funding EVAR satisfied the Publicity condition.

#### Appeals Condition

There was no formal means by which anyone could dispute or appeal the decisions made. Respondents suggested that if there was a concern, they could approach the Surgeon-in-Chief. There was a conflict between the radiologists and surgeons regarding which provider should perform EVAR. There was no external attempt to mediate this conflict. The hospital decision-maker became involved when funding became an issue. When asked if there was an appeals mechanism when it became apparent that funding would be cut, one surgeon explained:

"*We disputed it at multiple levels including with [Surgeon-in-Chief], with [CEO], with [Board Chair], with any Board member that would listen because we thought it was, in fact, such a travesty. We had patients and...all the while we were frankly hopeful there would be some sort of cash infusion. In fact, we ultimately took it to the Ministry when [CEO] would not act in any appreciable way*".

Based on this data, we found that neither the decision to adopt EVAR nor the decision to stop it satisfied the Appeals condition.

#### Enforcement Condition

There was no explicit mechanism or central body responsible for decision-making to enforce any of the conditions of *Accountability for Reasonableness *or equivalent concepts.

## Discussion

Surgical innovations are driven in large measure by a surgeon's desire to innovate and improve health care. Consequently, patients with major medical conditions who would have been deemed inoperable in the past are now candidates for interventions due to the decreased risk associated with some surgical innovations. On the other hand, patients face risks when new surgical technologies are introduced. There is a fine line between 'innovation' and 'experimentation'. Moreover, surgical innovations may have significant resource implications for individual institutions and the entire health care system. Therefore, there is a role for oversight of even well-intentioned decision-making by surgeons [28].

How a surgical technology is valued by a hospital depends on many factors. EVAR posed a large economic impact on the institution. The institution's views on the innovation and its effectiveness differed from that of the providers. The conflicting values evident in the decisions made affected the vascular surgery division, resulting in the recruitment and subsequent loss of a vascular surgeon. Furthermore, patients were exposed to a procedure with unestablished outcomes. Conflicting values existed between the surgeons and hospitals. Surgeons were interested in bringing the 'newest' and 'best' technologies to their patients, and did not describe being heavily influenced by their cost. Hospitals want to provide health services within their constrained resources. These conditions highlight the importance of developing a fair process for the evaluation of future innovations in surgery.

*Accountability for Reasonableness *is a framework that has been used to evaluate priority setting at both the hospital level and at the level of clinical programs such as cardiac surgery [29,30], but has not been used to focus specifically on decisions to adopt surgical innovations at the level of a surgeon or surgical department.

*Accountability for Reasonableness *provides an explicit framework to evaluate this process and describe good practices and recommendations for improvement that can be used to help inform the development of guidelines to aid decision-makers in the adoption of future surgical innovations at the hospital level.

The decision to adopt EVAR was driven by a few individuals interested in adopting the new technology focusing on a very narrow range of factors. Communication was made with a hospital decision-maker and the Research Ethics Board to inform them of the procedure prior to adoption, however other departments that were affected should have been engaged in this process (such as the radiology department). Communication occurred primarily among the members of the vascular surgery department. Greater involvement from administration to identify financial constraints would have been beneficial to help avoid stopping EVAR once it began. A key hospital decision-maker at the time was a vascular surgeon, which may have posed a conflict of interest in the decision. On the other hand, it is possible that this decision-maker had better knowledge about the value of EVAR at a time when other stakeholders were less enthusiastic about its value.

A mechanism to ensure competence was used by recruiting a surgeon from another hospital with prior experience with EVAR. This reduced the learning curve associated with acquiring a new skill for an innovation [31], presumably increasing the success and safety of the performance of the procedure. However, according to the *Accountability for Reasonableness *framework, the decision to adopt and the decision to stop EVAR were not made fairly.

The decision to adopt EVAR was based on perceived improved patient outcomes and safety and the surgeons' desire to innovate. The decision-making process responsible for this adoption followed mainly the "medical-individualistic" perspective [32] which focuses on hospitals adopting new technologies based on the clinical needs of their patient population and the benefit of the intervention for the patient. This decision is driven by the clinician or hospital medical administrator regardless of whether economic considerations or other factors suggest alternative decisions [33]. Greenberg et al (2005) found that most adoption decisions for new technologies come from senior physicians, but the responsibility for the final adoption decisions varies depending on the technology. For innovations that involve large capital investments, the final decision was usually made by the hospital's senior executives, or by ad hoc committees consisting of similar administrative and medical leaders [33].

There was very little medical evidence available in the literature at the time, and there were no long-term data available due to the novelty of the procedure. Based on the surgeon's desire to help their patients who they felt, in many cases, had few other options, this was seen as a logical choice and therefore offered to their patients. However, this decision involved few stakeholders – other departments that were affected were not engaged at the time. Furthermore, hospital decision-makers were not adequately informed, which later had an impact on the decision to stop funding the procedure.

The decision to stop funding involved all key players and was based on criteria apparent to all those involved, including cost, evidence and hospital priorities. In light of the conceptual framework used in this study, the initial decision to introduce the technology did not satisfy two of the conditions of *Accountability for Reasonableness*, however, the decision to terminate it did. It is interesting that a poor process can produce what, in retrospect, appeared to be a 'good' decision and that a 'good' process does not guarantee that a 'good' decision will be the result.

Several lessons can be learned from this analysis. In order to improve the decision-making process, hospitals should develop a structure for deliberating the reasons for adopting a surgical innovation. This recommendation is consistent with other studies of technology adoption at the hospital level where a centralized process should be established with a medical director involvement [33,34]. The process should involve a wide range of stakeholders including managers, a financial officer from the institution with oversight of hospital budget, some representation from the public, community member, department head and other departments affected to allow the full range of relevant considerations to be included. Furthermore, broader input should be sought, not solely from individuals personally invested in the innovation. A 'disinterested' person, one at 'arms length' to the advocates of the innovation, would have the appearance of being less biased in favour of its adoption.

Hospitals should also establish a formal appeals mechanism for addressing challenges to the decisions being made [35]. This may have facilitated discussions between radiologists and the surgeons, improving the quality of communication between key stakeholders. This should not be regarded as a dispute resolution mechanism as much as a mechanism for improving the quality of decisions being made (see Table 2). Structures that currently exist in hospitals that may act as an appeals mechanism might include department heads, advisory committees or ethics boards. While these individuals or groups may assist in such decisions, their role is not mandated or structured. To ensure consistency and fairness in such decision-making processes, it is important to establish a structure that can address such issues for surgeons and their teams.

**Table 2:** Recommendations for improving the decision-making process

1. Hospitals should develop a structure for deliberating the reasons for adopting a surgical innovation that involve a wide range of stakeholders.
2. Broader input should be sought from individuals involved with the procedure and those at "arms length" who may not be directly invested in the innovation.
3. Hospitals should establish a formal appeals mechanism for addressing challenges to the decisions being made.

Our study has several limitations. The results from this case study represent findings from an academic health sciences centre, which may not be generalizable to smaller community hospitals. The goal of qualitative research however, is to provide a rich description of a context-specific phenomenon derived from empirical research. It would be useful to study different types of surgical innovations using the same framework and in different settings. The small sample size may be considered a limitation, however, we interviewed all the individuals who were involved in the process at the time and those who had experience with the innovation. This small number represents all the key players as identified through our sampling strategy.

A challenge presented throughout the interview process was the respondents' recall of past events, and their perceptions at the time of implementation. Since we were asking opinions of events that occurred several years ago, some of the recalled details may be inaccurate. We did, however, obtain consistent information from the different respondents, in addition to document analyses that corroborated information from key informant interviews, suggesting that respondents' representations were valid.

## Conclusion

We described and evaluated the decision-making process for the adoption of a new surgical technology for the treatment of abdominal aortic aneurysms at a large, university teaching hospital in Toronto, Canada. Several recommendations were made to improve the decision-making process involved in adopting surgical innovations in hospitals. Results from this case study may apply to similar settings where a formal process for decision-making does not exist and where conflicting pressures such as professional autonomy, budgetary constraints, and strategic organizational planning affect individual surgeon's decisions and practices. We are using these empirical findings and observations from other case studies, analyzing a spectrum of surgical innovations of varying magnitudes across community and academic hospitals, to develop a framework for a decision-making process for the adoption of surgical innovations in Canadian hospitals.

## Competing interests

The author(s) declare that they have no competing interests.

## Authors' contributions

ND conducted the interviews, performed data analysis and drafted the manuscript. DM participated in the design of the study. PL participated in the design of the study and data analysis. JH participated in the interview process and data analysis. RS participated in the design of the study. MB participated in the design of the study. DU conceived of the study, participated in its design and coordination and helped draft the manuscript. All authors read, reviewed and approved the final manuscript.

## Pre-publication history

The pre-publication history for this paper can be accessed here:


